# Preferences and acceptability of law enforcement initiated referrals for people who inject drugs: a mixed methods analysis

**DOI:** 10.1186/s13011-020-00319-w

**Published:** 2020-10-02

**Authors:** Gabriella K. Olgin, Annick Bórquez, Pieter Baker, Erika Clairgue, Mario Morales, Arnulfo Bañuelos, Jaime Arredondo, Alicia Harvey-Vera, Steffanie Strathdee, Leo Beletsky, Javier A. Cepeda

**Affiliations:** 1 Division of Infectious Diseases and Global Public Health, University of California,San Diego, La Jolla, CA USA; 2 Graduate School of Public Health, San Diego State University, San Diego, CA USA; 3 School of Government and Public Policy, University of Arizona, Tucson, AZ USA; 4 Department of Planning and Special Projects, Secretaría de Seguridad Pública Municipal, Tijuana, Mexico; 5 Centro de Investigación y Docencia Económica, Aguascalientes, Mexico; 6 School of Law and Bouve College of Health Sciences, Northeastern University, Boston, MA USA; 7 Department of Epidemiology, Johns Hopkins Bloomberg School of Public Health, Baltimore, MD USA

**Keywords:** People who inject drugs, Law enforcement, Harm reduction, Referral

## Abstract

**Background:**

Law enforcement officers (LEOs) come into frequent contact with people who inject drugs (PWID). Through service referrals, LEOs may facilitate PWID engagement in harm reduction, substance use treatment, and other health and supportive services. Little is known about PWID and LEO attitudes and concerns about service referrals, however. The objective of this mixed-methods study was to examine the alignment of service referral preferences and acceptability among PWID and LEOs in Tijuana, Mexico.

**Methods:**

We assessed service referral preferences and perceived likelihood of participation in health and social services, integrating data from structured questionnaires with 280 PWID and 306 LEOs, contextualized by semi-structured interviews and focus groups with 15 PWID and 17 LEOs enrolled in two parallel longitudinal cohorts in Tijuana, Mexico.

**Results:**

Among potential service referral options, both PWID (78%) and LEOs (88%) most frequently cited assistance with drug- and alcohol-use disorders. Over half of PWID and LEOs supported including harm reduction services such as syringe service programs, overdose prevention, and HIV testing. The majority of PWID supported LEO referrals to programs that addressed basic structural needs (e.g. personal care [62%], food assistance [61%], housing assistance [58%]). However, the proportion of LEOs (30–45%) who endorsed these service referrals was significantly lower (*p* <  0.01). Regarding referral acceptability, 71% of PWID reported they would be very likely or somewhat likely to make use of a referral compared to 94% of LEOs reporting that they thought PWID would always or sometimes utilize them. These results were echoed in the qualitative analysis, although practical barriers to referrals emerged, whereby PWID were less optimistic that they would utilize referrals compared to LEOs.

**Conclusions:**

We identified strong support for LEO service referrals among both LEO and PWID respondents, with the highest preference for substance use treatment. LEO referral programs offer opportunities to deflect PWID contact with carceral systems while facilitating access to health and social services. However, appropriate investments and political will are needed to develop an evidence-based (integrated) service infrastructure.

## Introduction

Drug law enforcement practices (e.g. syringe confiscation, arrest) have been shown to increase public health risks, such as HIV transmission [1–7]. This has been evidenced in numerous global settings such as the United States; Kiev, Ukraine; Bangkok, Thailand; and Tijuana, Mexico [8–12]. Recently, there has been growing support to reform drug laws, including efforts to decriminalize personal consumption of drug use and support harm reduction and rehabilitation efforts [13].

A shift toward more effective measures to address drug-related harms has also included changes in law enforcement practice. Law enforcement officers (LEOs) are being increasingly called upon to refer people who use drugs to care, to administer naloxone to individuals with suspected opioid overdoses, and to use enforcement discretion to promote public health [14–20]. Some evidence suggests LEOs’ attitudes and behaviors may align with public health goals, particularly in settings that are expanding harm reduction in response to the North American overdose crisis [16, 17]. Experienced LEOs recognize the importance of social services and their limited availability and accessibility [18]. Additionally, LEOs often regard arrest as an ineffective instrument for addressing drug-related harms, especially in the era of COVID-19 [18, 21]. Rather than punitive approaches, police-assisted referrals to drug treatment and other services might be more effective from a public health standpoint [15]. Nevertheless, the traumatic legacy of drug law enforcement, with disproportionate burden on communities of color, raises questions about the acceptability and appropriateness of police-assisted treatment navigation and other health-focused efforts [22].

In 2009, the Mexican government enacted reforms (“narcomenudeo”), in part, to treat low level drug offenses as a public health problem rather than a criminal justice one. Among other provisions, the reform decriminalized possession of small amounts of illicit drugs at the federal level; however, implementation was not observed at the state and municipal levels [23, 24]. Border cities like Tijuana, Mexico, are important hubs of drug trafficking, injection drug use, and HIV [25, 26], which are endemic among key populations such as people who inject drugs (PWID). The drug treatment resources that currently exist in Tijuana and other parts of Mexico range from abstinence-based centers that often violate human rights to voluntary, evidence-based methadone maintenance clinics [27]. To reduce visibility of drug use as part of the city’s beautification efforts, LEOs have previously referred PWID to services [28]. However, these referrals have been limited to forced referrals to compulsory abstinence programs [27]. Such referrals and other negative interactions between LEOs and PWID have been shown to increase HIV risk among PWID [4, 11, 26, 29]. These negative interactions, along with the failure to implement the 2009 drug law reform at the municipal level, prompted the development of a police education program in Tijuana. The *Escudo* (“Shield” in Spanish) police training program aimed to train LEOs on enforcing the reforms’ stipulations to align policing practices with public health, including referral of PWID to harm reduction services [30, 31].

While there have been numerous police-assisted referral programs in the United States [14, 15, 18, 19], there is little known about preferences stated by the intended beneficiaries, PWID, or about the alignment between LEO’s and PWID’s attitudes towards these measures. Successful implementation and adoption of a police referral programs requires careful consideration of the attitudes and intended behaviors of both LEOs and PWID. To that end, we conducted a mixed-methods analysis among these two key groups in Tijuana, Mexico, to examine the alignment of service referral preferences and acceptability of a potential police referral program to reduce drug-related harms.

## Methods

We adopted an explanatory mixed-methods approach with qualitative data used to contextualize and explain quantitative findings [32].

### Quantitative methods

We used cross-sectional data from two longitudinal cohorts, *El Cuete* and *Escudo*, which were conducted among PWID and LEOs in Tijuana, respectively. Procedures for both studies have been described previously [31, 33]. Briefly, recruitment for *El Cuete* was initiated in Tijuana from March 2011 to May 2013 with additional recruitment occurring from April 2016 to April 2018. In total, 734 and 209 PWID were recruited over the two recruitment periods, respectively. The referral measures relevant to this analysis were added to the survey in January 2018, resulting in an analytic sample size of 280 participants who completed these survey items. The eligibility criteria for the study included: aged 18 years or older, having injected illicit drugs within the past month confirmed by inspection of injection stigmata, ability to speak Spanish or English, and willingness to provide informed consent. Trained staff recruited participants in places which PWID frequented. A targeted sampling scheme was used to maximize both the safety of the participants and staff, as well as to promote the recruitment of female participants.

Regarding *Escudo* recruitment procedures, 1806 LEOs received a three-hour police education program between February 2015 and May 2016 [31]. The program focused on three main training goals: (1) occupational safety related to needlestick injuries and risk of HIV/hepatitis C virus transmission, (2) Mexican drug policy reforms and legality of syringe possession, and (3) drug pharmacology and public health benefits of harm reduction services. Inclusion criteria were aged 18 years or older, being an active LEO, and willingness to consent to study procedures. Participants self-administered pre- and post-training surveys that reviewed concepts discussed in the training, attitudes on harm reduction services, and frequency of behaviors leading to increased HIV risk among PWID (e.g. confiscating syringes, making arrests for syringe possession, throwing away syringes in the trash) in the past 6 months. A random sample of 707 officers were followed for 2 years. Since the referral items were added to the survey halfway through the 24-month (final) follow-up visit, the analytical sample consisted of 306 officers.

#### Measures and analysis

##### PWID survey

We collected sociodemographic data and quantitatively assessed referral service preferences and likelihood of attending referral services. Participants were requested to indicate their preference (yes/no) for each of the following referral services: assistance with drug- and alcohol-use disorders; HIV or other infectious disease testing; HIV treatment; syringe service programs; overdose prevention; wound care and other health care; dental clinic; food assistance; legal or immigration assistance; housing assistance; employment assistance; laundry, showers, or other personal care; other services; or none/would not accept assistance from police. Likelihood of attending the referral services was also assessed using a 4-point Likert scale ranging from *very likely* to *not likely*, which we dichotomized to likely/very likely and unlikely/very unlikely.

##### LEO survey

The items included in the Escudo survey had been drawn from similar training evaluation initiatives in other settings [34, 35]. The survey questions were piloted among senior level LEOs and their feedback was incorporated in the final version of the instrument. Because of this, there were slight differences in some of the answer choices between the PWID and the LEO surveys. Similar to the referral locations asked among PWID, LEOs indicated their preferences regarding the following services: assistance with drug- and alcohol-use disorder; HIV or other infectious disease testing; overdose prevention; basic health care; food assistance; legal or immigration assistance; housing assistance; employment assistance; laundry, showers, or other personal care; other services. LEOs also indicated how likely they thought PWID would be to attend the referral services on a 4-point Likert scale. This variable was dichotomized to sometimes/all the time and rarely/never. Differences between the PWID and LEO responses were analyzed using the chi-squared test. We compared responses from questions that had similar choices. As our goal was to compare responses across both samples, any choices that were not present in both surveys were excluded from the analysis.

### Qualitative methods

To explain people’s referral service preferences, we also conducted semi-structured, in-depth qualitative interviews and focus groups.

#### Semi-structured in-depth interviews with PWID

We selected a convenience sample of 15 PWID (14 men and 1 woman) participating in the *El Cuete* cohort. Eligibility criteria included having been incarcerated in Tijuana in the past 6 months for more than 30 days in order to capture participants that had previously been exposed to LEOs and would be able to better relate to the concept of referral to services instead of incarceration. Interviews took place between March 2017 and July 2018, until saturation of main themes was reached. We conducted the interviews in Spanish, which lasted between 60 and 90 min. We asked participants about past experiences of referral to services by LEOs, views regarding the feasibility of a police referral intervention through the use of vouchers, perceptions related to police referral to services addressing drug use or health problems and about services that would be useful to them in the context of this intervention. We transcribed the interviews verbatim and fidelity of transcription was assessed against the audio-records. We assessed English translations for consistency and accuracy. Participation in the study was voluntary, anonymous and respondents received 20 USD for participation.

#### Focus groups among LEOs

We conducted three focus groups in June 2018, totaling 17 LEOs. Four district level chiefs, three males and one female, participated in the first focus group while the second and third focus groups consisted of street level officers (7 males and 6 females) in districts with high illicit drug use. The focus groups included discussion of perceptions about PWID, HIV/HCV prevention services and referrals, facilitators and barriers for referrals and harm reduction facilities, as well as feasibility and acceptability of such programs. Each focus group was moderated by two members of the research team and lasted approximately 90 min. All participants consented to study procedures and received a 20 USD gift card. Audio recordings were transcribed verbatim and translated to English.

#### Qualitative data analysis

We developed an initial scheme of codes based on the interview guide and followed a deductive approach to qualitative data analysis. We used Atlas.ti v.8.3 and applied thematic analysis to identify new themes and generated new codes accordingly [36]. We matched and integrated lists of codes into a single codebook and assessed inter-rater reliability. We resolved disagreements in their assignment/description through discussion among coders and iteratively modified codes accordingly to finalize the coding structure. We present illustrative quotes with pseudonyms.

#### Ethical approval

Study protocols for all quantitative and qualitative studies were approved by the Institutional Review Boards of the University of California, San Diego School of Medicine and Universidad Xochicalco, Facultad de Medicina, Campus Tijuana as well as the Ethics Board of the Colegio de la Frontera Norte, Tijuana.

## Results

### Overview of study sample

A total of 586 participants were included in the quantitative analyses, 280 and 306 of whom were PWID and LEOs, respectively (See Table 1). Of the 280 PWID, 40% were female, and the mean age was 44.5 (standard deviation (sd): 8.9 years). Thirty-six percent of PWID reported earning less than 3500 pesos (185 USD) per month over the past year and 83% reported using heroin in the last 6 months. Two percent of PWID were living with HIV. Approximately 40% were stopped at least once by the police and 12% were arrested in the last 6 months. Nearly 20% of PWID reported being forced to pay a bribe to police in the last 6 months. Among the sample of LEOs, the mean age was 38.5 (sd: 8.5 years) and most of the LEOs were male (89%). Approximately one third (29%) of the LEOs worked in a high drug use district along the Tijuana River Canal (i.e. Centro, La Mesa, Mesa de Otay). The majority served as patrol officers (84%) and the mean number of years of experience was 12.8 (sd: 7.8 years). All officers reported frequently or sometimes coming into contact with needle and syringes while working.

**Table 1:** Characteristics of the PWID sample (*n* = 280) and LEO sample (*n* = 306)

Characteristics	n (%) or mean ± SD
PWID sample	
Age	44.5 ± 8.9
Female	111 (40%)
Used heroin in the last 6 months	232 (83%)
Earned < 3500 pesos (185 USD) per month in the last year	100 (26%)
Living with HIV	6 (2%)
Stopped by police in the last 6 months	116 (41%)
Arrested in the last 6 months	33 (12%)
Forced to pay a bribe to police in the last 6 months	52 (19%)
LEO sample	
Age	38.5 ± 8.5
Female	34 (11%)
Worked in high drug use district along Tijuana River Canal	89 (29%)
Patrol officers	257 (84%)
Years of experience	12.8 ± 7.8
Frequently came into contact with needles/syringes during work	96 (31%)

*PWID* people who inject drugs, *LEO* law enforcement officers

### Referral to drug treatment and harm reduction

Overall, 78% of PWID reported that they would like treatment/rehab centers included in a referral program (See Table 2). However, qualitative findings suggest that overall views on treatment centers were mixed and setting-specific. In the qualitative interviews, participants often reported mistreatment occurring at these centers.

I: “Have you been referred to a center?”

P: “Oh yes, but it was a really bad one, where they beat you, they tie you up, the food is really bad, only squash soup and people are horrible. You suffer here in the streets to then go to a center where you’re gonna be humiliated, abused, treated like garbage, for what? You feel undermined, you get out with lower self-esteem, it’s like you don’t feel like the person you are, you’re made to feel less, instead of coming out with a clearer mind, you come out with an even more messed up mind.” (Diego, PWID)

Conversely, others said they would accept a voucher or coupon from the officer when making the referral to attend evidence-based treatment programs, such as a methadone program.

I: “And what would you think of a referral to a methadone program? If the police officer gave you a voucher to start a methadone program?”

P: “To start methadone? I would say yes, but it should be well administered you know? Because personally I don’t think you need to be on methadone for months or weeks to get over heroin, you need to take it for 5 days at most and with that you don’t get withdrawal.” (Mateo, PWID)

Overall, LEOs were supportive towards drug rehabilitation. Significantly more LEOs (88%) than PWID (78%) reported assistance with drug- and alcohol-use disorders as a service they would want included (*p* < .01), but in the focus groups the LEOs highlighted the need for higher quality rehabilitation centers and resources. Some officers specifically noted the public health importance of harm reduction to reduce infectious disease transmission and expressed interest in establishing a relationship with programs such as syringe service programs or safe injection sites.

“Look, if there was what we could call a free rehab center, I would much rather take any given person out of circulation and, instead of putting them in jail, send them to the center where I know for sure they will remain for a six-month treatment. That would be better for me. It would mean one less person going around infecting or committing crime in my area. It would be preferable to put them in a free rehab center for six months so that, when they are released, they can be a little more cured, so to speak.” (Jose, LEO)

“Condoms and syringes. To me, as a public servant, that is great. They are telling them, ‘You are already sick, that’s the truth, but protect yourself and don’t go spreading (the disease) around.” (Lucia, LEO)

“A place where they can inject and have a doctor available to do it properly. That would be much better. A single area… A booth for them to inject would really help us, where they can exchange their needles. A place where they can shoot up would be good.” (Luis, LEO)

**Table 2:** Referral service preferences of people who inject drugs and law enforcement officers in Tijuana, Mexico

	PWID n (%)	LEOs n (%)	***p***-value
**Drug treatment and harm reduction**			
Addiction assistance	218 (78%)	270 (88%)	0.001
Overdose prevention	165 (59%)	173 (57%)	0.59
Syringe service program	168 (60%)	161 (53%)	0.08
**Basic needs**			
Laundry, showers, or other personal care	174 (62%)	133 (43%)	< 0.001
Food assistance	172 (61%)	114 (37%)	< 0.001
Housing assistance	162 (58%)	93 (30%)	< 0.001
Legal or immigration assistance	144 (51%)	98 (32%)	< 0.001
Employment assistance	180 (64%)	191 (62%)	0.68
Basic health care^a^	174 (62%)	171 (56%)	0.14
HIV or other infectious disease testing	163 (58%)	206 (67%)	0.02
HIV treatment	165 (59%)	N/A^b^	N/A
Dental clinic	131 (47%)	N/A^b^	N/A
**Likelihood the PWID would attend the referral service**^**c**^			
Very likely/All the time	136 (49%)	184 (60%)	N/A
Somewhat likely/Sometimes	61 (22%)	105 (34%)	N/A
Somewhat unlikely/Rarely	39 (14%)	13 (4%)	N/A
Very unlikely/Never	42 (15%)	2 (1%)	N/A

*PWID* people who inject drugs, *LEOs* law enforcement officers

^a^This question was worded differently in the survey among PWID (“wound care or other health care”) than in the survey among LEOs (“basic health care”)

^b^Not asked in the survey among LEOs

^c^Responses for PWID ranged from Very unlikely to Very likely and for LEOs ranged from Never to All the time

### Referral programs for basic needs

PWID often cited services beyond drug rehabilitation programs to address substance use problems. They were significantly more supportive of police referrals to programs that addressed basic needs (e.g. personal care [62% vs. 43%], food assistance [61% vs. 37%], housing assistance [58% vs. 30%], legal/immigration assistance [51% vs. 32%]) than LEOs (*p* <  0.001). In qualitative interviews, referrals to food and personal care services were viewed positively by some PWID and with reluctance by others, as they felt these would not solve their underlying issues. Employment and legal assistance were viewed as being critical to reduce reliance on drugs and to support reintegration into society.

"Mmmm. Well, a shower, a bath. A haircut. Things like that too, do you know what I mean? Things that the person can also use to get money… Clothes too, not just food. They [Police] should also offer work, to clean, to sweep, things like that." (Miguel, PWID)

I: “If for example they could refer you to a place where they can help you find a job, would that be useful?”

P: “To me it would, it would, because actually, I want, and I will get my ID card. I don’t know how I’m gonna do it, but I’m gonna go to the town hall, I think they charge about 300 pesos, but I’m gonna go get my ID and get a job, maintenance, security, whatever, I don’t know, but I’ll get a job and from then on I know things will change. I want to work, but I don’t have an ID and they won’t take me anywhere without the paperwork.” (Juan, PWID)

While significantly fewer LEOs cited referral programs which addressed the basic needs, several officers nonetheless recognized the value of these types of programs as an important component of rehabilitation.

“…I would really like this and I’ve brought up this project because I have been attending a Christian church. These assistance or information centers would also take them to portable restrooms so they can shower… That is motivation for them, to give them a t-shirt or garment. One that’s more dignified than the one they’re wearing. I think that would really help these people.” (Jorge, LEO)

### Acceptability of referral program

Although our quantitative findings suggest PWID had a relatively high acceptance of police referral (71% of PWID reported that would be very likely or somewhat likely to use a police referral), mistrust of LEOs was common. Due to this mistrust, there were PWID who were skeptical about the feasibility of a referral program and the motives of the police. Nonetheless, not all PWID were concerned about an officer-initiated referral program and the acute need for services often superseded their mistrust in the police.

“Oh man! This guy [LEO]! I don’t know, he comes from another planet, what the fuck. It’s just that it’s very strange for a cop to do something like that [referral]…a cop will always fuck you. That's why, I don’t know, if changes like that started happening, it would be amazing, it’s gonna be great, I think. There’ll be addicts [who want a better life], who don’t know how to stop, or don’t know how to change and don’t have opportunities like that, those will grab those opportunities. They will find a door for a better future...” (Maria, PWID)

LEOs expressed frustration with the current ways of policing and thus were supportive of the implementation of a referral program.

“…for addicts not to regard us as just the ones who arrest them and take them to jail. I mean, for them to approach us, because all that will also help them. “I have this problem, officer. Where can I check in to or where can you send me?” “Well, you can go here, they will be able to help you there,” or just talk to the colleagues who are more well-versed on the matter, all that information, for them to inform and help them, because we don’t do anything outside of just sending them to jail, incessantly taking them to and from jail. “Hey, this person did this, go and take them again and drop them off again.” I mean, all that needs to stop.” (Paulina, LEO)

## Discussion

To our knowledge, this is the first analysis to specifically report input from PWID in conjunction with LEOs on referral preferences and acceptability of an LEO-initiated referral program. Overall, our findings suggest that the implementation of voluntary, police-led referral programs in Tijuana could be accepted by police and PWID. The strongest alignment of preferences among PWID and LEOs was on harm reduction services, with assistance for drug- and alcohol-use disorders being cited most frequently. However, most rehabilitation centers in Tijuana are non-evidence based, and several PWID reported negative experiences in terms of both being referred to these centers against their will and of mistreatment received while at the center [27, 37]. Additionally, PWID frequently cited the need for services addressing basic needs such as hygiene, medical care, and access to employment opportunities, while LEOs usually did not prioritize these services. Thus, addressing basic needs could be a key component of a police-initiated referral which might also improve access and retention to harm reduction services.

Amidst calls for drug decriminalization and reform of prohibitionist drug policy [13], police will be critical agents in the successful implementation of such reforms. These reforms may include substituting punitive tasks (e.g. arrest for drug possession for personal use) with public health-oriented tasks, such as administering naloxone to opioid overdoses [17]. Indeed, serving as a first responder also opens the opportunity for referral to treatment and other health services [16]. According to provisions in the Mexican drug policy reforms, referral to drug treatment is mandated upon the third violation of a drug possession infraction. However, because many of the drug treatment programs are not evidence-based and referrals are often involuntary, this mandate may be inadvertently exacerbating violation of human rights and public health harms [37, 38].

While establishing a voluntary, PWID-centered police referral program would be novel in Mexico, the concept of police referring people who use drugs has already been implemented in other North American settings. For example, the Law Enforcement Assisted Diversion (LEAD) program which was initiated in Seattle, Washington, has been active since 2011 [14]. Consistent with our findings, senior level officers strongly supported the LEAD referral program [14]. However, street level officers were more hesitant to refer people who use drugs to programs which did not require abstinence. Officers in Seattle remarked on the futility of arrest, which we also documented [14]. Furthermore, after involvement in the program, participants had significantly lower odds of being arrested or charged with a felony [15]. The city of Baltimore is also piloting the LEAD program and an initial survey of police officers found that a majority of their officers would report PWID to social or syringe service programs [18]. Additionally, few police officers believed current policing strategies were effective or that arrest efficiently reduced drug use [18]. Similarly, LEOs in Tijuana found current policing strategies ineffective at reducing drug use [28].

Other police departments in the United States have focused on a self-referral program, where PWID can voluntarily seek treatment navigation assistance from police. Evidence from Gloucester, Massachusetts, suggests that the current drug treatment system is fragmented and may be a barrier to retention after referral. However, most participants were already engaged in recovery or treatment programs. A self-referral program might be theoretically practical in Tijuana; however, mistrust toward the police will need to be overcome for PWID to voluntarily approach officers. In addition, previous analyses from our study found that LEOs were often reluctant to refer PWID due to fear of being accused of kidnapping [28]. While both PWID and LEOs reported a relatively high acceptance of a theoretical referral program, there is currently no system in place to facilitate voluntary referrals. Thus, it is unknown how frequently referrals would be accepted in a real-world situation. There is a strong need and desire for a harm reduction infrastructure among both PWID and LEOs in Tijuana, but for this vision to deliver expectations, large investments and political will are needed to ensure transparency and respect in the referral process.

An important finding was that PWID differed from LEOs in the interpretation of referral to drug treatment. Notably, PWID often discussed their concern that currently available drug treatment centers frequently commit violence and other human rights abuses [37, 39]. Conversely, LEOs often considered hypothetical treatment centers that would be regulated and funded by the government. Indeed, publicly funded methadone clinics do exist, however LEOs were often unaware of them. While referral to existing evidence-based programs, such as methadone programs, was often met with approval from PWID, there are numerous barriers to access, including cost and limited knowledge about such programs [30, 40]. LEOs were more optimistic about the likelihood that PWID would attend the referral services than PWID themselves, possibly because LEOs considered only hypothetical effective treatment centers rather than those currently available. This might also explain why significantly fewer PWID reported that they would accept a referral to these sites, as many had experienced abuse in these settings. Additionally, the antagonistic relationship between LEOs and PWID, which is exacerbated by the high rate of LEOs forcing PWID to pay bribes, has reinforced trauma and mistrust. Thus, including incentives for successful referral could potentially reverse this.

PWID consistently mentioned referral to programs to address basic needs (e.g. showers/clean clothes) and structural barriers including possessing IDs and finding employment and housing. Basic needs have been shown to be integral to the recovery and rehabilitation of PWID [41, 42], however LEOs usually did not discuss programs addressing this. Thus, it is likely that addressing substance use related problems should be prioritized as a referral service yet bundled with services to address basic needs in order to garner support and utilization from LEOs and PWID. Despite LEOs being more optimistic about acceptability of a referral program than PWID, 66% of PWID nonetheless reported that they would visit these referral services.

This study has several limitations. Generalizability of study findings might be limited because data were collected from one city in Mexico that is unique due to its proximity to the United States. Selection bias might also be a concern as participants were mostly derived from a convenience sample and individuals who did not participate could have held different views than those who were included. PWID who participated to the qualitative interviews had been incarcerated for over 30 days in the past 6 months, which may have negatively affected their perception of LEOs, while also conferring them a better understanding of the potential implications of a law enforcement-initiated referral system in the context of the drug law reform. Secondly, responses from LEOs might be vulnerable to social desirability since surveys and interviews were conducted within the context of a research study focusing on aligning policing with public health. While members of research staff conducted the focus groups which could have elicited socially desirable responses, the quantitative surveys were self-administered which might have reduced this inclination. Additionally, some responses could not be compared directly due to slight differences in the wording of the questions after piloting the surveys within each cohort. Finally, only one woman participated in the in-depth interviews with PWID. We have previously found female PWID can experience different acts of sexual and physical violence from police in Tijuana compared to male PWID [43]. As such, we acknowledge the importance of conducting more qualitative research among female PWID to better understand the acceptability and feasibility of a police-led referral system.

## Conclusion

Despite a legacy of trauma and frayed relations, an unexpectedly high proportion of PWID reported that they would accept referrals from LEOs. Reframing the role of LEOs in Tijuana as agents of change who can make a difference in their community by facilitating access to services among PWID is the ultimate goal. Indeed, pathways to deflect PWID to services can be achieved in theory, but investment is needed to ensure that these services are available, easily accessible and of high quality. Implementation of such referral programs could be mutually beneficial in that LEOs could divert resources to more serious crimes while PWID would have the opportunity to address their medical and basic needs.

## Data Availability

The data analyzed in the current study are not publicly available due to confidential and potentially sensitive information. Data are available from the corresponding author on request.

## Abbreviations

HCV: Hepatitis C virus
HIV: Human immunodeficiency virus
LEAD: Law enforcement assisted diversion
LEO: Law enforcement officer
PWID: People who inject drugs
